# Real-life effectiveness of montelukast administered as monotherapy or in combination with inhaled corticosteroid (ICS) in pediatric patients with uncontrolled asthma

**DOI:** 10.1186/1710-1492-10-S1-A55

**Published:** 2014-03-03

**Authors:** Denis Bérubé, Michel Djandji, John S Sampalis, Allan Becker

**Affiliations:** 1 Centre Hospitalier Universitaire (CHU) Sainte-Justine, Université de Montréal, Montréal, Québec, H7M 5M2, Canada; 2 Merck Canada Inc., Kirkland, Québec, H9H 3L1, Canada; 3 McGill University, Montréal, Québec, H3G 1Y6, Canada; 4 JSS Medical Research, St-Laurent, Québec, H4S 1N8, Canada; 5 Section of Allergy and Clinical Immunology, Department of Pediatrics and Child Health, University of Manitoba, Winnipeg, Manitoba, R3A 1S1, Canada

## Background

The efficacy of montelukast in the treatment of asthma has been demonstrated in numerous controlled clinical trials. The aim of this study was to assess the real-life effectiveness of montelukast administered as monotherapy or in combination with current ICS in children with uncontrolled asthma.

## Methods

Twelve-week open-label, phase IV, multicenter, prospective cohort study. Eligible patients included children aged 2-14 years diagnosed with asthma for =6 months who were: (i) uncontrolled as per the Canadian Asthma Consensus Guidelines, and; (ii) either untreated, using a short-acting ß2–agonist as-needed or using any dose ICS. In this analysis, patients with Asthma Control Questionnaire (ACQ) score >0.75 were included. Patients 6-14 and 2-5.9 years old were treated once-daily with montelukast 5mg and 4mg, respectively. Primary outcome measure was the proportion achieving asthma control (ACQ=0.75). Secondary outcomes were the absolute change in ACQ and in the Pediatric Asthma Caregiver’s Quality of Life Questionnaire (PACQLQ) over time.

## Results

Among the 328 patients included, 76 (23.2%) were treated with montelukast monotherapy and 252 (76.8%) with montelukast combined with ICS. By 4 weeks of treatment 61.3% and 52.9% of patients in the monotherapy and combination group, respectively, achieved asthma control. These proportions increased to 75.0% and 70.9%, respectively, at week-12. Clinically and statistically (P<0.001) significant improvements were observed in ACQ (monotherapy: mean (SD) of 1.67 (0.69) at baseline and 0.50 (0.52) at week-12; combination therapy: 2.02 (0.83) and 0.64 (0.86), respectively) and PACQLQ (monotherapy: mean (SD) of 5.34 (1.14) at baseline and 6.51 (0.85) at week-12; combination therapy: 4.42 (1.35) and 6.21 (1.03), respectively) in both patient subgroups. After a 12-week montelukast add-on therapy, 22.6% of patients reduced their ICS dosage.

## Conclusions

Montelukast as monotherapy or in combination with ICS represents an effective treatment strategy for achieving asthma control in pediatric patients and improving caregivers’ quality of life.

## Acknowledgements

On behalf of the ACTION^3^ investigators.

## Trial registration

Clinicaltrials.gov: 00832455.

